# Ethyl 4-(2-fur­yl)-2-oxochroman-3-carboxyl­ate

**DOI:** 10.1107/S1600536810016193

**Published:** 2010-05-12

**Authors:** Maddela Prabhakar, J. S. N. Reddy, N. Ravi Kumar, S. Viswanadha Ganesh, K. Anand Solomon

**Affiliations:** a Institute of Life Sciences, University of Hyderabad Campus, Hyderabad 500 046, India

## Abstract

The title compound, C_16_H_14_O_5_, was prepared from the reaction of 3-carbethoxy­coumarin with furan in the presence of AlCl_3_ as catalyst. In the crystal, inter­molecular C—H⋯O hydrogen-bonding inter­actions between four mol­ecules lead to a tetra­mer in the unit cell. The furan ring is anti­periplanar [C—C—C—O = 167.9 (13)°] and the ethoxy­carbonyl group is (−)anti­clinal [C—C—C—O = −128.6 (14)°] to the lactone ring.

## Related literature

For the medicinal and biological activity of coumarins and their derivatives, see: Borges *et al.* (2005 ▶); Kontogiorgis & Hadjipavlou-Litina (2005 ▶); Gursoy & Karali (2003 ▶); Prabhakar *et al.* (2010 ▶). For the assignment of conformations and the orientation of the substituents, see: Nardelli (1983 ▶, 1995 ▶); Klyne & Prelog (1960 ▶).
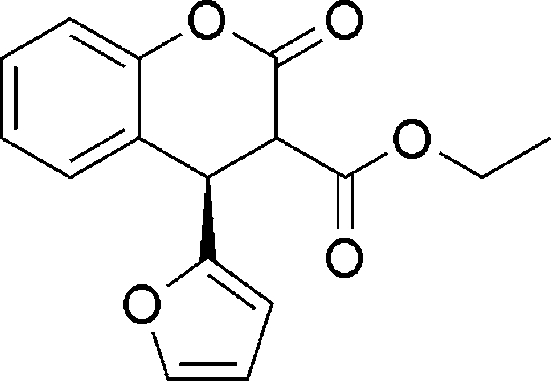


## Experimental

### 

#### Crystal data

C_16_H_14_O_5_
*M* *_r_* = 286.27
Monoclinic, 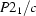

*a* = 10.393 (3) Å
*b* = 8.459 (3) Å
*c* = 15.819 (5) Å
β = 95.464 (5)°
*V* = 1384.5 (8) Å^3^
*Z* = 4
Mo *K*α radiation
μ = 0.10 mm^−1^
*T* = 298 K
0.34 × 0.24 × 0.20 mm

#### Data collection

Bruker SMART CCD area-detector diffractometer
Absorption correction: multi-scan (*SADABS*; Bruker, 2003 ▶) *T*_min_ = 0.966, *T*_max_ = 0.980
13767 measured reflections
2711 independent reflections
2099 reflections with *I* > 2σ(*I*)
*R*_int_ = 0.035

#### Refinement

*R*[*F* ^2^ > 2σ(*F* ^2^)] = 0.042
*wR*(*F* ^2^) = 0.111
*S* = 1.04
2711 reflections
227 parameters
H atoms treated by a mixture of independent and constrained refinement
Δρ_max_ = 0.18 e Å^−3^
Δρ_min_ = −0.16 e Å^−3^

### 

Data collection: *SMART* (Bruker, 2003 ▶); cell refinement: *SAINT* (Bruker, 2003 ▶); data reduction: *SAINT*; program(s) used to solve structure: *SHELXS97* (Sheldrick, 2008 ▶); program(s) used to refine structure: *SHELXL97* (Sheldrick, 2008 ▶); molecular graphics: *SHELXTL* (Sheldrick, 2008 ▶); software used to prepare material for publication: *SHELXTL*.

## Supplementary Material

Crystal structure: contains datablocks I, global. DOI: 10.1107/S1600536810016193/ds2028sup1.cif

Structure factors: contains datablocks I. DOI: 10.1107/S1600536810016193/ds2028Isup2.hkl

Additional supplementary materials:  crystallographic information; 3D view; checkCIF report

## Figures and Tables

**Table 1:** Hydrogen-bond geometry (Å, °)

*D*—H⋯*A*	*D*—H	H⋯*A*	*D*⋯*A*	*D*—H⋯*A*
C3—H3⋯O3^i^	0.972 (15)	2.696 (15)	3.576 (2)	150.8 (11)
C16—H16*B*⋯O2^ii^	0.96	2.70	3.549 (3)	148
C16—H16*A*⋯O2^iii^	0.96	2.96	3.841 (3)	153
C8—H8⋯O3^iv^	0.96 (2)	2.94 (2)	3.611 (3)	128.2 (15)
C11—H11⋯O4^v^	0.93 (2)	2.73 (2)	3.501 (3)	140.9 (17)
C13—H13⋯O2^vi^	1.01 (2)	2.54 (2)	3.456 (3)	151.0 (17)
C12—H12⋯O4^vii^	0.95 (2)	2.74 (2)	3.478 (3)	134.9 (16)

Symmetry codes: (i) 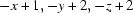; (ii) 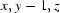; (iii) 
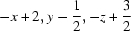
; (iv) 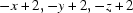; (v) 
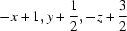
; (vi) 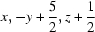; (vii) 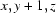.

**Fig. 1.**
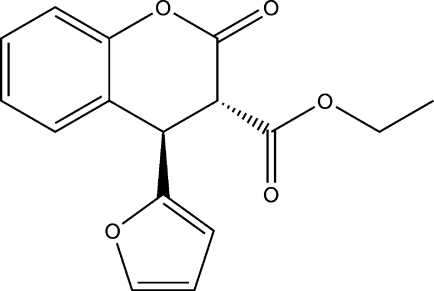
Chemical diagram of the title compound.

**Fig. 2.**
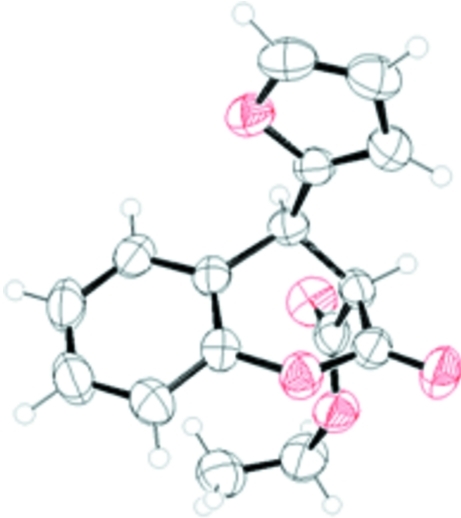
ORTEP diagram of the 3-carbethoxy-4-(2-furyl)-chroman-2-one. (Thermal ellipsoids are at 50% probability level).

**Fig. 3.**
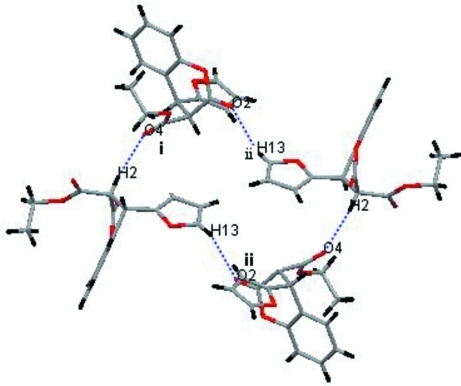
Crystal packing of (I) showing the formation of tetramer. The C—H&bull;&bull;&bull;O contacts are shown as dashed lines. Symmetry code: (i) 1-x,-1/2+y, 1/2-z (ii) x, -1/2-y, -1/2+z

**Fig. 4.**
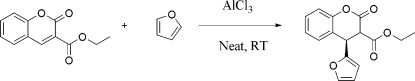
The formation of the title compound.

## supplementary crystallographic information

### Comment

We have synthesized and reported our serendipitous observations on the Diels-Alder reaction of 3-carbethoxy coumarin with furan, followed by a ring opening to yield Michael product, 3-carbethoxy-4-(2-furyl)-chroman-2-one in good yields.

### Experimental

3-Carbethoxy coumarin (3 m mol) was taken into 30 mmol of furan and 10 mol% of AlCl_3_ catalyst was added. The reaction mixture was stirred at room temperature for 24 hours. After completion of the reaction, the excess of the furan was distilled off and extracted thrice with water/dichloromethane. The product was separated from flash column chromatography and recrystallized from dichloromethane.

### Refinement

All H atoms were found on difference maps, with C—H=0.93 Å and included in the final cycles of refinement using a riding model, with Uiso(H)=1.2Ueq(C)

### Crystal data

**Table:**

C_16_H_14_O_5_	*F*(000) = 600
*M**_r_* = 286.27	*D*_x_ = 1.373 Mg m^−^^3^
Monoclinic, *P*2_1_/*c*	Mo *K*α radiation, λ = 0.71073 Å
Hall symbol: -P 2ybc	Cell parameters from 5174 reflections
*a* = 10.393 (3) Å	θ = 2.5–25.8°
*b* = 8.459 (3) Å	µ = 0.10 mm^−^^1^
*c* = 15.819 (5) Å	*T* = 298 K
β = 95.464 (5)°	Block, colourless
*V* = 1384.5 (8) Å^3^	0.34 × 0.24 × 0.20 mm
*Z* = 4	

### Data collection

**Table:**

Bruker SMART CCD area-detector diffractometer	2711 independent reflections
Radiation source: fine-focus sealed tube	2099 reflections with *I* > 2σ(*I*)
graphite	*R*_int_ = 0.035
phi and ω scans	θ_max_ = 25.9°, θ_min_ = 2.0°
Absorption correction: multi-scan (*SADABS*; Bruker, 2003)	*h* = −12→12
*T*_min_ = 0.966, *T*_max_ = 0.980	*k* = −10→10
13767 measured reflections	*l* = −19→19

### Refinement

**Table:**

Refinement on *F*^2^	Primary atom site location: structure-invariant direct methods
Least-squares matrix: full	Secondary atom site location: difference Fourier map
*R*[*F*^2^ > 2σ(*F*^2^)] = 0.042	Hydrogen site location: inferred from neighbouring sites
*wR*(*F*^2^) = 0.111	H atoms treated by a mixture of independent and constrained refinement
*S* = 1.03	*w* = 1/[σ^2^(*F*_o_^2^) + (0.058*P*)^2^ + 0.1619*P*] where *P* = (*F*_o_^2^ + 2*F*_c_^2^)/3
2711 reflections	(Δ/σ)_max_ < 0.001
227 parameters	Δρ_max_ = 0.18 e Å^−^^3^
0 restraints	Δρ_min_ = −0.15 e Å^−^^3^

### Fractional atomic coordinates and isotropic or equivalent isotropic displacement parameters (Å^2^)

**Table:**

	*x*	*y*	*z*	*U*_iso_*/*U*_eq_	
H2	0.5893 (16)	0.9788 (19)	0.7769 (10)	0.045 (4)*	
H13	0.648 (2)	1.325 (3)	1.0884 (14)	0.084 (7)*	
H11	0.598 (2)	1.251 (2)	0.8313 (14)	0.076 (6)*	
H12	0.598 (2)	1.472 (3)	0.9435 (13)	0.081 (6)*	
H7	1.082 (2)	0.672 (3)	1.0318 (14)	0.085 (7)*	
H6	0.8933 (19)	0.630 (2)	1.0962 (13)	0.073 (6)*	
H8	1.077 (2)	0.828 (2)	0.9093 (13)	0.078 (6)*	
H5	0.6973 (18)	0.7462 (19)	1.0400 (11)	0.048 (5)*	
H3	0.5753 (15)	0.8783 (17)	0.9186 (9)	0.033 (4)*	
C6	0.8921 (2)	0.6956 (2)	1.04771 (12)	0.0626 (5)	
O3	0.65502 (12)	1.13244 (14)	1.01627 (7)	0.0545 (3)	
O1	0.88812 (11)	0.98037 (14)	0.83531 (7)	0.0523 (3)	
O5	0.74734 (13)	0.71312 (14)	0.73664 (7)	0.0598 (4)	
O2	0.78518 (13)	1.08381 (15)	0.72090 (7)	0.0635 (4)	
C3	0.65067 (15)	0.93666 (18)	0.90397 (9)	0.0404 (4)	
O4	0.57089 (13)	0.66307 (15)	0.80304 (8)	0.0651 (4)	
C4	0.77275 (15)	0.86004 (17)	0.94453 (9)	0.0388 (4)	
C1	0.77894 (17)	1.00313 (19)	0.78254 (10)	0.0460 (4)	
C9	0.88546 (15)	0.88312 (18)	0.90688 (10)	0.0429 (4)	
C2	0.65772 (16)	0.92533 (19)	0.80739 (10)	0.0425 (4)	
C14	0.65204 (17)	0.7516 (2)	0.78227 (10)	0.0465 (4)	
C13	0.63873 (19)	1.2923 (2)	1.02691 (14)	0.0597 (5)	
C8	1.00056 (18)	0.8154 (2)	0.93811 (12)	0.0576 (5)	
C5	0.77720 (19)	0.7640 (2)	1.01532 (10)	0.0502 (4)	
C10	0.63742 (14)	1.10518 (19)	0.93083 (9)	0.0419 (4)	
C11	0.61161 (18)	1.2410 (2)	0.89003 (12)	0.0550 (5)	
C12	0.61250 (19)	1.3618 (2)	0.95281 (13)	0.0593 (5)	
C15	0.7673 (2)	0.5456 (2)	0.71929 (12)	0.0718 (6)	
H15A	0.8029	0.5339	0.6652	0.086*	
H15B	0.6853	0.4902	0.7162	0.086*	
C7	1.0035 (2)	0.7225 (2)	1.00924 (13)	0.0656 (5)	
C16	0.8571 (2)	0.4771 (2)	0.78765 (14)	0.0738 (6)	
H16A	0.9356	0.5376	0.7935	0.111*	
H16B	0.8763	0.3698	0.7737	0.111*	
H16C	0.8177	0.4795	0.8401	0.111*	

### Atomic displacement parameters (Å^2^)

**Table:**

	*U*^11^	*U*^22^	*U*^33^	*U*^12^	*U*^13^	*U*^23^
C6	0.0812 (15)	0.0569 (11)	0.0475 (10)	0.0109 (10)	−0.0047 (10)	0.0124 (9)
O3	0.0658 (8)	0.0534 (7)	0.0449 (7)	0.0049 (6)	0.0093 (5)	−0.0084 (5)
O1	0.0463 (7)	0.0581 (7)	0.0526 (7)	−0.0034 (5)	0.0059 (5)	0.0137 (6)
O5	0.0819 (9)	0.0466 (7)	0.0524 (7)	0.0094 (6)	0.0151 (7)	−0.0060 (5)
O2	0.0824 (9)	0.0614 (8)	0.0474 (7)	0.0061 (7)	0.0110 (6)	0.0171 (6)
C3	0.0377 (8)	0.0423 (9)	0.0416 (8)	−0.0022 (7)	0.0052 (7)	−0.0030 (7)
O4	0.0666 (8)	0.0592 (8)	0.0684 (9)	−0.0165 (7)	0.0007 (7)	−0.0116 (6)
C4	0.0459 (9)	0.0342 (8)	0.0357 (8)	−0.0005 (6)	0.0000 (6)	−0.0043 (6)
C1	0.0571 (10)	0.0409 (9)	0.0403 (9)	0.0072 (7)	0.0058 (8)	0.0004 (7)
C9	0.0453 (9)	0.0417 (8)	0.0410 (8)	−0.0012 (7)	0.0005 (7)	0.0016 (7)
C2	0.0446 (9)	0.0423 (9)	0.0392 (8)	0.0078 (7)	−0.0041 (7)	−0.0021 (7)
C14	0.0539 (10)	0.0482 (9)	0.0352 (8)	0.0012 (8)	−0.0077 (7)	−0.0034 (7)
C13	0.0629 (12)	0.0556 (11)	0.0622 (12)	−0.0001 (9)	0.0144 (10)	−0.0197 (10)
C8	0.0456 (10)	0.0652 (12)	0.0609 (11)	0.0047 (8)	−0.0008 (9)	0.0030 (9)
C5	0.0623 (11)	0.0471 (10)	0.0420 (9)	−0.0002 (8)	0.0081 (8)	0.0010 (8)
C10	0.0384 (8)	0.0479 (9)	0.0394 (8)	0.0024 (7)	0.0044 (6)	−0.0057 (7)
C11	0.0628 (12)	0.0512 (10)	0.0508 (11)	0.0106 (8)	0.0039 (9)	−0.0008 (9)
C12	0.0629 (12)	0.0441 (10)	0.0723 (13)	0.0059 (9)	0.0138 (10)	−0.0066 (9)
C15	0.1093 (17)	0.0474 (11)	0.0597 (12)	0.0141 (11)	0.0133 (12)	−0.0108 (9)
C7	0.0622 (13)	0.0673 (13)	0.0641 (12)	0.0171 (10)	−0.0104 (10)	0.0071 (10)
C16	0.0813 (15)	0.0552 (11)	0.0863 (15)	0.0111 (10)	0.0158 (12)	0.0064 (11)

### Geometric parameters (Å, °)

**Table:**

C6—C7	1.377 (3)	C2—C14	1.522 (2)
C6—C5	1.381 (3)	C2—H2	0.937 (17)
C6—H6	0.94 (2)	C13—C12	1.317 (3)
O3—C10	1.3663 (19)	C13—H13	1.01 (2)
O3—C13	1.375 (2)	C8—C7	1.371 (3)
O1—C1	1.357 (2)	C8—H8	0.96 (2)
O1—C9	1.4020 (19)	C5—H5	0.962 (18)
O5—C14	1.321 (2)	C10—C11	1.333 (2)
O5—C15	1.462 (2)	C11—C12	1.425 (3)
O2—C1	1.1969 (19)	C11—H11	0.93 (2)
C3—C10	1.498 (2)	C12—H12	0.95 (2)
C3—C4	1.512 (2)	C15—C16	1.478 (3)
C3—C2	1.539 (2)	C15—H15A	0.9700
C3—H3	0.972 (15)	C15—H15B	0.9700
O4—C14	1.197 (2)	C7—H7	0.95 (2)
C4—C9	1.378 (2)	C16—H16A	0.9600
C4—C5	1.381 (2)	C16—H16B	0.9600
C1—C2	1.506 (2)	C16—H16C	0.9600
C9—C8	1.375 (2)		
			
C7—C6—C5	120.05 (18)	O3—C13—H13	113.0 (13)
C7—C6—H6	120.5 (13)	C7—C8—C9	118.97 (19)
C5—C6—H6	119.4 (13)	C7—C8—H8	119.9 (13)
C10—O3—C13	106.29 (14)	C9—C8—H8	121.0 (13)
C1—O1—C9	120.03 (12)	C6—C5—C4	120.55 (18)
C14—O5—C15	117.97 (15)	C6—C5—H5	122.2 (10)
C10—C3—C4	112.56 (13)	C4—C5—H5	117.2 (10)
C10—C3—C2	110.80 (13)	C11—C10—O3	109.59 (14)
C4—C3—C2	106.09 (12)	C11—C10—C3	134.68 (15)
C10—C3—H3	108.5 (8)	O3—C10—C3	115.73 (13)
C4—C3—H3	110.1 (8)	C10—C11—C12	107.06 (17)
C2—C3—H3	108.7 (8)	C10—C11—H11	124.6 (13)
C9—C4—C5	118.03 (15)	C12—C11—H11	128.3 (13)
C9—C4—C3	117.99 (13)	C13—C12—C11	106.68 (17)
C5—C4—C3	123.94 (15)	C13—C12—H12	126.2 (13)
O2—C1—O1	118.36 (16)	C11—C12—H12	127.1 (13)
O2—C1—C2	124.98 (16)	O5—C15—C16	109.51 (16)
O1—C1—C2	116.65 (13)	O5—C15—H15A	109.8
C8—C9—C4	122.14 (15)	C16—C15—H15A	109.8
C8—C9—O1	116.82 (15)	O5—C15—H15B	109.8
C4—C9—O1	121.02 (13)	C16—C15—H15B	109.8
C1—C2—C14	111.48 (13)	H15A—C15—H15B	108.2
C1—C2—C3	110.60 (13)	C8—C7—C6	120.24 (18)
C14—C2—C3	108.36 (13)	C8—C7—H7	120.9 (14)
C1—C2—H2	105.5 (10)	C6—C7—H7	118.8 (14)
C14—C2—H2	108.9 (10)	C15—C16—H16A	109.5
C3—C2—H2	112.1 (10)	C15—C16—H16B	109.5
O4—C14—O5	125.46 (16)	H16A—C16—H16B	109.5
O4—C14—C2	122.91 (16)	C15—C16—H16C	109.5
O5—C14—C2	111.62 (15)	H16A—C16—H16C	109.5
C12—C13—O3	110.37 (17)	H16B—C16—H16C	109.5
C12—C13—H13	136.6 (13)		

### Hydrogen-bond geometry (Å, °)

**Table:**

*D*—H···*A*	*D*—H	H···*A*	*D*···*A*	*D*—H···*A*
C3—H3···O3^i^	0.972 (15)	2.696 (15)	3.576 (2)	150.8 (11)
C16—H16B···O2^ii^	0.96	2.70	3.549 (3)	148
C16—H16A···O2^iii^	0.96	2.96	3.841 (3)	153
C8—H8···O3^iv^	0.96 (2)	2.94 (2)	3.611 (3)	128.2 (15)
C11—H11···O4^v^	0.93 (2)	2.73 (2)	3.501 (3)	140.9 (17)
C13—H13···O2^vi^	1.01 (2)	2.54 (2)	3.456 (3)	151.0 (17)
C12—H12···O4^vii^	0.95 (2)	2.74 (2)	3.478 (3)	134.9 (16)

Symmetry codes: (i) −*x*+1, −*y*+2, −*z*+2; (ii) *x*, *y*−1, *z*; (iii) −*x*+2, *y*−1/2, −*z*+3/2; (iv) −*x*+2, −*y*+2, −*z*+2; (v) −*x*+1, *y*+1/2, −*z*+3/2; (vi) *x*, −*y*+5/2, *z*+1/2; (vii) *x*, *y*+1, *z*.
